# Blood Transfusion Frequency and Indications in Yemeni Children with Sickle Cell Disease

**DOI:** 10.1155/2020/7080264

**Published:** 2020-08-24

**Authors:** Abdul-Wahab M. Al-Saqladi, Dikra M. Maddi, Aida H. Al-Sadeeq

**Affiliations:** ^1^Pediatric Department, Faculty of Medicine and Health Sciences, University of Aden, Aden, Yemen; ^2^Pediatric Department, Al-Sadaqa Teaching Hospital, Aden, Yemen

## Abstract

**Background:**

Blood transfusion is an essential component in the care of patients with sickle cell disease (SCD), but it might be associated with serious acute and delayed complications. This study was aimed to describe red cell transfusion patterns and indications among hospitalized SCD children in a low-resource setting. *Patients and Methods.* A retrospective, descriptive study of all children (≤16 years) with SCD who received blood transfusion therapy during their hospital admissions in the pediatric department at Al-Sadaqa Teaching Hospital in Aden, Yemen, for a period of one year.

**Results:**

Out of 217 hospitalized children with SCD, 169 (77.9%) were transfused and received 275 RBC transfusion episodes. The mean age of transfused children was 6.9 ± 4.6 years and 103 (60.9%) were males, with a male/female ratio of 1.6 : 1 (*p*=0.004). Hemoglobin (Hb) levels were significantly lower in the transfused than in the nontransfused (Hb 5.5 ± 1.5 vs. 7.7 ± 1.5 g/dL, *p*=0.03). Pretransfusion Hb levels were ˂7.0 g/dL in 86.2% and ˂5.0 g/dL in 39.3% of patients. Single transfusion was given to 122 (72.2%) and 5 or more transfusions in 9 (4.15%) of patients on different occasions. Simple (top-up) transfusion was used in all transfusion events. Commonest indications for transfusion were anemic crises (41.1%), vasoocclusive crises (VOC) (13.8%), VOC with anemic event (11.3%), acute chest syndrome (8.7%), and stroke (7.3%).

**Conclusion:**

Intermittent blood transfusion remains a common practice for the management of children with acute SCD complications. Main indications were acute anemic crises, severe pain crises, ACS, and stroke. In limited resource settings, such as Yemen, conservative transfusion policy appears to be appropriate.

## 1. Introduction

Blood transfusion is a key component in the comprehensive care of patients with sickle cell disease (SCD), and it can alleviate symptoms, reduce complications, and improve patient quality of life [1]. Blood transfusion can be lifesaving when used correctly, but it might be associated with serious acute and delayed complications [2]. Red blood cells (RBC) transfusion in all recipients has a potential risk of infection transmission, transfusion reactions, iron overload, alloimmunization, and acute and delayed hemolytic transfusion reactions (DHTRs) [3].

In patients with SCD, transfusion of normal RBCs is intended to correct the oxygen-carrying capacity, diluting circulating sickle RBCs, and suppress their endogenous production, reducing the proportion of sickle hemoglobin (HbS) concentration, with subsequent improvement in blood viscosity and microcirculatory perfusion [4].

Intracellular polymerization of deoxygenated HbS leads to alteration of shape and physical properties of red blood cells, which become rigid, more fragile, and easily destructed, resulting in shortened of sickle erythrocyte lifespan and chronic anemia [5]. Although moderate anemia is tolerated at steady state, further worsening can cause a relative anemia, which is a serious frequent event, with a variety of short-term and long-term detrimental consequences and usually require prompt transfusion therapy [6].

Intermittent RBC transfusion is indicated for episodes of acute anemia occurring with splenic sequestration, transient aplastic crisis, hyperhemolytic crisis, and other acute complications such as acute chest syndrome (ACS), acute stroke, acute sickle hepatopathy, multisystem organ failure, and perioperative management [7]. Chronic transfusion is indicated for the primary and secondary prevention of stroke, recurrent splenic sequestration, recurrent ACS, and frequent refractory painful crises that do not response to hydroxyurea therapy [8].

Blood transfusion can be administered by a simple (top-up) or exchange transfusion. Simple transfusion is more frequently used for acute transfusion, as it is more convenient and easier to administer, while exchange transfusion (automated or manual) is used in case of higher pretransfusion hemoglobin, at risk of hypervolemia, and in clinical conditions requiring rapid reduction in the proportion of HbS and hyperviscosity [9].

In patients with African haplotypes, hemoglobin level in steady state is usually between 6.0 and 9.0 g/dL, compared with slightly higher level of 8.0–11.0 g/dL in the Asian haplotype [10]. In Yemen, the predominant *β*^s^ haplotypes are Benin (82.6%) and followed by Bantu (5.6%), with nonexistence of the Asian haplotype [11]. In a previous study, the average hemoglobin level in Yemeni SCD children at steady state was found to be 7.4 ± 1.4 g/dL [12].

There is an increasing interest on the role of transfusion therapy in the management of SCD, and the utilization of RBC transfusion is expanding, with an estimation that over 50.0% of SCD children would have received at least one or more blood transfusion in their pediatric life time [13, 14].

Children with SCD in our locality are frequently transfused [15], but detailed information about transfusion practice are not available. This study is aimed to present our experience on RBC transfusion patterns and indications among hospitalized SCD children at Hematology/Oncology Center in Aden, South Yemen.

## 2. Methods

### 2.1. Study Design

This is a retrospective, descriptive study of all children with sickle cell disease who received blood transfusion therapy as part of their clinical management during their hospital admission into the pediatric medical department at Al-Sadaqa Teaching Hospital, for the period of one year (January–December 2014).

### 2.2. Setting

The study site was the Hematology/Oncology Center at pediatric department in Al-Sadaqa Teaching Hospital, which is the main pediatric and maternity hospital in Aden, South Yemen. This hospital is a public health facility serving people from Aden and adjacent governorates and provides medical care for the majority of pediatric patients with SCD in this locality. The study included all transfused children (≤16 years) with a clinical diagnosis of SCD supported by a positive sickling test and confirmed by Hb electrophoresis. Medical care for admitted children including blood transfusion was carried out by an experienced clinical team and according to the hospital norms. Transfusion decision was based on the level of Hb ˂5.0 g/dL, acute reduction of Hb more than 2.0 g/dL than the baseline, and according to the clinical status of the patient and the presence of complications. The entire pediatric blood transfusions in our department are performed in the in-patient setting. In almost all circumstances, blood collection was depending on the replacement donors who were recruited by patients' relatives. Screening for HIV, HBV, and HCV are carried out routinely in our blood bank before any blood donation, and positive blood donors for aforementioned infections are excluded before collection.

### 2.3. Data Collection

All medical records of hospitalized SCD children over one-year period were reviewed thoroughly, and relevant information was extracted to a standardized data abstraction form. For each patient, the following variables were noted: age, sex, number of transfusions, indication or immediate reason triggering transfusion events, pretransfusion hemoglobin level, volume and method of transfusion, type and duration of transfusion, premedication, adverse transfusion reactions, and hydroxyurea therapy.

### 2.4. Definitions

A transfusion episode was defined as any blood transfusion within a 48-hour period of hospital stay. Notable indications included painful vasoocclusive crisis (VOC), ACS (appearance of a new infiltrate on chest X-ray, associated with one or more of clinical symptoms such as chest pain, respiratory distress, fever, and cough), stroke (onset of acute neurologic symptoms and signs lasting for 1 day), and acute febrile illness, which indicates infection without clear focus, lower respiratory tract infection, and acute dactylitis (hand-foot syndrome). Anemic crises including hemolytic crisis, acute splenic sequestration crisis, and transient red cell aplasia were considered altogether as we could not specify each type with adequate certainty and to avoid events misclassifications.

### 2.5. Ethical Clearance

Investigators have accessed the patients' information from their medical records, by a permission obtained from the hospital administration. Informed consent was not required since the study was retrospective and performed by a chart review without any potential risk implicated upon the patients. Confidentiality, deidentification, and anonymity of personal data were strictly maintained all throughout.

### 2.6. Statistical Analysis

The data collected were analyzed using SPSS for Windows version 20. Descriptive statistics were presented as frequency and percentage of various clinical characteristics. Quantitative variables were presented as mean and SD or median and range as appropriate. Data were analyzed using the chi-square test or Student's *t*-test, and the level of significance was set as *p* value less than 0.05.

## 3. Results

Out of 217 hospitalized SCD children, 169 (77.9%) were transfused and 48 (21.1%) were not transfused during the 12 months of study period. Among the transfused children, 103 (60.9%) were males and 66 (39.1%) were females, giving a male/female ratio of 1.6 : 1 (*p*=0.004). The mean age of transfused children was 6.9 ± 4.6 years, while that of nontransfused was 8.0 ± 4.7 years, with no significant differences (*p*=0.16). Among the transfused children, age group 1–5 years was most commonly encountered (40.8%) and received the highest proportion (38.2%) of transfusions, but the highest transfusion rate (1.8/patient) was noted in the age band 6–10 years (Table 1).

**Figure 1:**
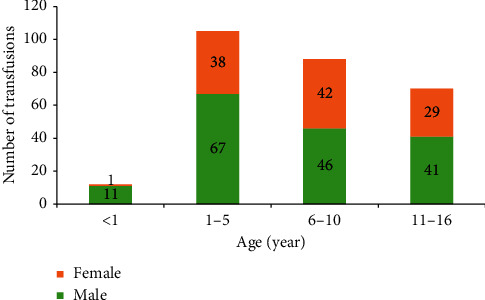
Frequency of transfusion episodes by age and gender.

Transfusion frequency by age groups and gender is shown in Figure 1. Males received 169 and females 110 transfusions, with a rate per patient of 1.6 and 1.7, respectively.

The frequency of transfusion events for all patients during the whole study period is depicted in Table 2. Among 169 transfused children, single transfusion was given to 122 (72.2%) patients, 2 transfusions in 22 (13.0%), 3 transfusions in 11 (6.5%), 4 transfusions in 5 (3.0%) of patients, and 5 times or more occurred in 9 (4.1%) patients.

**Figure 2:**
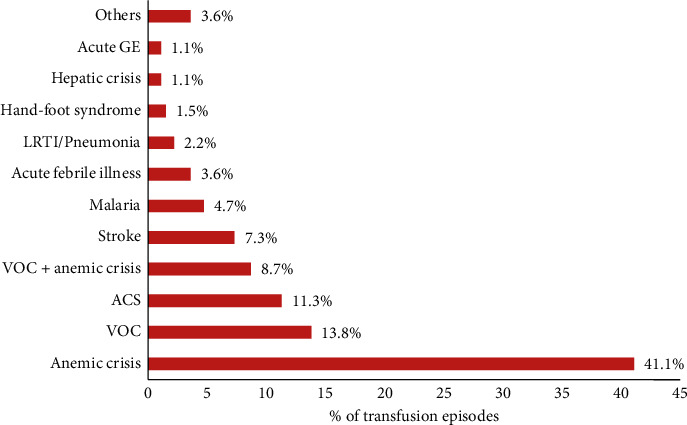
Distribution of 275 transfusion episodes according to indications.

The indications for blood transfusion in the current study are summarized in Table 3 and Figure 2. Anemic crisis accounted for 113 (41.1%) transfusions in 76 patients with a mean Hb level of 4.2 ± 0.9 g/dL (range 2.6–6.0). VOC accounted for 38 (13.8%) transfusions in 31 patients with a mean Hb level of 7.0 ± 1.0 g/dL (range 4.1–8.7), while VOC associated with anemic crisis occurred in 21 patients and accounted for 24 (8.7%) transfusions with a mean Hb of 5.2 ± 0.2 g/dL (range 5.0–5.3). ACS accounted for 31 (11.3%) transfusions in 28 patients with a mean Hb of 6.2 ± 1 g/dL (range 3.6–8.1). Five patients presented with overt stroke and received 20 (7.3%) transfusions with a mean Hb of 6.5 ± 0.8 g/dL (range 6.0–7.7). There were 13 patients with documented malaria who received 13 (4.7%) transfusions with a mean Hb of 5.6 ± 1.5 g/dL (range 5.3–8.5). Acute febrile illness was the indication for 10 (3.6%) transfusions in 10 patients with a mean Hb of 5.6 ± 0.6 g/dL (range 4.3–6.3).

The overall average of hemoglobin levels in hospitalized SCD children was 6.0 ± 1.8 g/dL, and it was significantly lower in the transfused than in the nontransfused (Hb 5.5 ± 1.5 vs. 7.7 ± 1.5 g/dL, *p*=0.03). Mean pretransfusion Hb levels in both males and females were almost equal 5.5 ± 1.5 vs. 5.5 ± 1.0 g/dL.

Out of the 275 transfusion episodes, 108 (39.3%) patients had hemoglobin levels below 5.0 g/dL, 129 (46.9%) between 5.0 and 6.9 g/dL, 25 (9.1%) between 7.0 and 7.9 g/dL, and 13 (4.7%) between 8.0 g/dL and 8.9 g/dL (Table 4).

Simple (top-up) transfusion was used in all transfusion events, and blood was slowly infused usually within a duration of 2–4 hours. Prophylactic administration of loop diuretics such as furosemide injection before transfusion was only given on individual basis, at risk of circulatory overload and cardiopulmonary complications. Almost all transfusion types were of packed RBCs and calculated in volume for body weight (usual dose 15.0 ml/kg).

Three cases had probable transfusion reactions. Allergic reaction was developed in 2 cases (a 5 year female and an 8 year male) who had a history of previous transfusions. As well as one case of febrile reaction in a 16 year female, transfused for the first time. All reactions were of mild type and brought under control by medications including antihistamine, corticosteroid, and antipyretics. Alloimmunization testing, blood group subtyping, and leukoreduction of transfused blood are not available in our hospital. Hydroxyurea (HU) treatment was reported in three cases, two of them with overt stroke and used the drug as secondary prophylaxis.

## 4. Discussion

This study is the first of its kind to look at the utilization rates and indications of blood transfusion in the management of hospitalized children with SCD in Yemen. Among the 217 hospitalized SCD children, 77.9% received blood transfusion therapy as part of their in-patient management care. Transfusion frequency in our sample is comparable to those reported by others, 80.6% from Congo [16] and 73.8% from Iraq [17]. However, our frequency rates were higher than the 31.0% reported from Oman [18] and 39.1% in Nigeria [19] but lower than 90.3% reported in a Tanzanian study [20]. Variations in transfusion requirements can be affected by genetic and environmental factors, as well as by ready availability of safe blood and effective transfusion services [21]. The influence of pretransfusion Hb levels at the time of admission as a main trigger of transfusion is likely to explain at least part of the differences in transfusion frequency rates. In our center, the overall Hb average of hospitalized SCD children was 6.0 g/dL, and more than three quarters of transfused children have Hb level less than 7.0 g/dL. Comparing with a study from India [22], our results showed that Hb levels in transfused patients were less than 7.0 g/dL in 86.2% vs. 75.7%, respectively, and 39.3% of our children have a higher rate of severe anemia (Hb ˂ 5.0 g/dL) compared to 23.3% in their sample. It is already known that patients with Asian haplotypes have higher Hb levels than those with African haplotypes [10].

Simple transfusion is the most widespread method of transfusion used in acute sickle cell events because it is commonly available, easy to perform, and technically feasible with minimal requirements of equipment and staff training [23]. While exchange transfusion is the preferable method in condition with an emergent need to decrease HbS concentration, in higher pretransfusion Hb (˃9.0 g/dL), and for chronic transfusion with increased risk of iron overload, but this method is technically demanding, and it requires special equipment, trained personnel, adequate blood supply, and usually a central venous access [7]. In this study, majority of children are suffering from relative anemia, and no transfusion was given to any patient with Hb level ≥9.0 g/dL.

Currently in our center, simple transfusion is the only option and appears to be appropriate, as exchange transfusion is not available and chronic transfusion program is not employed due to lack of facilities. Most families of our patients with overt stroke are reluctant to use regular transfusion as prophylaxis, even by a simple transfusion method because of its overall burden, potential risks of infections, iron overload, indefinite use, and cost of iron chelating therapy. In addition, a primary stroke prophylaxis is not possible with absence of transcranial Doppler (TCD) ultrasonography screening. Transfusion practice in SCD is variable worldwide, and results of an international survey showed that patients with SCD are often transfused intermittently, and chronic blood transfusion is less commonly practiced, particularly in the Middle East region and Africa [24].

The most common indications for transfusion in our study were anemic crises, VOC with/without anemic crises, ACS, and stroke. Detailed information about transfusion practice in SCD patients was coming from a Jamaican experience cohort study [25], and despite the differences in the nature of study design, hematological aspects might be comparable, given that the African Benin *β*^s^ haplotype is predominant in both Jamaican (76.0%) and Yemeni (82.0%) sickle cell patients [11, 26]. Comparing our results with that of the Jamaican study with regards to commonest indications showed anemic crises represented 41.1% vs. 41.7% (aplastic + sequestration + hypoplasia), ACS (11.3% vs. 17.3%), stroke (7.3% vs. 11.9%); whereas the average Hb levels were in anemic crises (4.2 vs. 4.0 g/dL), ACS (6.2 vs. 5.0 g/dL), and in stroke (6.5 vs. 6.3 g/dL), respectively. In the American cooperative study of SCD including a cohort follow-up in the first decade of life, the most common indications for acute transfusion were acute anemia (34.6%), ACS (27.5%), infection (9.8%), and surgery (9.8%) [27]. A recent study from the national comparative blood transfusion audit in UK reported the commonest reasons for urgent transfusions in SCD children to be anemia (30%) and acute ACS (18%) [28].

In this study, transfusions were given to 25 (9.1%) patients for VOC associated with exacerbation of anemia and to 38 (13.8%) with severe and prolonged VOC. One controversial issue is the use of RBC transfusion for the treatment of acute VOC, as currently, there is insufficient evidence for its benefit, and transfusion is not recommended unless there is an additional indication [7, 8]. In spite of this, transfusion for acute pain in clinical practice is continuing and even increasing likely because therapy options for this indication are limited, and transfusion is widely available and relatively safe [29, 30].

Hydroxyurea is a therapeutic agent used in SCD patients with potential multiple benefits and has been shown to reduce episodes of VOC, ACS, and the requirement for blood transfusions [9, 23]. Only three of our patients received HU treatment; this marked underutilization can be explained by the lack of ability to offer the drug free of charge and the overall burden of maintaining regular clinical and laboratory monitoring.

ACS is the leading cause of morbidity and mortality in patients with SCD and primarily managed by supportive therapy, antibiotics, and simple RBC transfusion [7]. As part of their clinical care, all patients with ACS in this study were given simple blood transfusions, which accounted for 11.3% of all transfusion episodes, a result located in the middle between 7.0% and 16.0% reported in Indian and Jamaican studies, respectively [22, 25].

Documented malaria infection in this sample was reported in 13 patients and accounted for 4.7% of transfusion episodes. Malaria in Yemen is of unstable endemicity, and antimalaria prophylaxis is not given routinely to children with SCD. Malaria remains a major cause of morbidity and mortality in sickle cell children living in endemic areas and is considered to be a common precipitant of pain and hyperhemolytic crises [6].

Among children with repeated transfusions, particularly in short intervals, some cases with anemic crises are assumed to develop antigenic disparities with alloimmunization and DHTRs. However, a low rate is expected due to the fact that in our hospital, blood donors are of the same ethnic/racial origin.

The main limitation of this study is the inability to discriminate types of anemic events due to the lack of diagnostic facilities. Since the data were collected retrospectively, detailed information about previous history of clinical events, in particular, the number of blood transfusions and its age of onset, was not consistently documented in the clinical records. Further studies are required to discern different types of anemic episodes, to determine prevalence of alloimmunization, and to explore effective measures, which could help in improvement of transfusion safety, reducing cost, and avoidance of unnecessary transfusion.

## 5. Conclusions

Simple (top-up) blood transfusion is a common practice in the management of children with SCD in our hospital. Majority of transfused children were anemic at the time of admission and more than one-third had severe anemia. Main indications for intermittent RBC transfusions were acute anemic crises, severe pain crises, ACS, and stroke. An improved utilization of hydroxyurea may lead to decrease in frequency of transfusion in this population. In limited resource settings, such as Yemen, maintaining a safe and adequate blood supply is challenging; therefore, conservative transfusion policy appears to be appropriate.

## Figures and Tables

**Table 1:** Distribution of transfused children and transfusion episodes by age and sex.

Variable	Children (*n* = 169)		Transfusion episodes (*n* = 275)		Rate/patient
	*n*	%	*n*	(%)	
Age (year)					
<1	9	5.3	12	(4.4)	1.3
1–5	69	40.8	105	(38.2)	1.5
6–10	50	29.6	88	(32.0)	1.8
11–16	41	24.3	70	(25.4)	1.7

Sex					
Male	103	60.9	165	(60.0)	1.6
Female	66	39.1	110	(40.0)	1.7

**Table 2:** Frequency of 275 transfusion episodes among 217 children with SCD.

No. of transfusions	Patients		Total transfusion events
	*n*	(%)^*∗*^	
0	48	—	—
1	122	72.2	122
2	22	13.0	44
3	11	6.5	33
4	5	3.0	20
5	4	2.3	20
6	2	1.2	12
7	2	1.2	14
10	1	0.6	10
Total	217	100.0	275

^*∗*^Those transfused.

**Table 3:** Indications for 275 blood transfusion episodes.

Indication	Transfusions		Number of patients/indication^*∗*^
	Number	Percentage	
Anemic crisis	113	41.1	76
VOC	38	13.8	31
ACS	31	11.3	28
VOC + anemic crisis	24	8.7	21
Stroke	20	7.3	5
Malaria	13	4.7	13
Acute febrile illness	10	3.6	10
LRTI/pneumonia	6	2.2	6
Hand-foot syndrome	4	1.5	3
Hepatic crisis	3	1.1	3
Acute GE	3	1.1	3
Others^†^	10	3.6	10
Total	275	100.0	209

^*∗*^Patient may had more than one indication on different occasions. ^†^Preoperative (2), UTI (2), hematuria (1), leg ulcer (1), osteomyelitis (1), hepatitis A (1), rheumatic heart disease (1), and kerosene poisoning (1).

**Table 4:** Pretransfusion hemoglobin level of transfused patients in 275 episodes.

Hemoglobin (g/dL)	Female	Male	All	
	*n*	*n*	*n*	(%)
2–2.9	4	8	12	(4.4)
3–3.9	15	18	33	(12.0)
4–4.9	20	43	63	(22.9)
5–5.9	37	45	82	(29.8)
6–6.9	23	24	47	(17.1)
7–7.9	6	19	25	(9.1)
8–8.9	5	8	13	(4.7)
Total	110	165	275	(100.0)

## Data Availability

Data used in this study are available from the corresponding author upon reasonable request.
